# Controlled Human Malaria Infection with Graded Numbers of *Plasmodium falciparum* NF135.C10- or NF166.C8-Infected Mosquitoes

**DOI:** 10.4269/ajtmh.18-0194

**Published:** 2018-07-16

**Authors:** Marijke C. C. Langenberg, Linda J. Wammes, Matthew B. B. McCall, Else M. Bijker, Geert-Jan van Gemert, Wouter Graumans, Marga G. van de Vegte-Bolmer, Karina Teelen, Cornelis C. Hermsen, Rob Koelewijn, Jaap J. van Hellemond, Perry J. J. van Genderen, Robert W. Sauerwein

**Affiliations:** 1 Institute for Tropical Diseases, Harbour Hospital, Rotterdam, The Netherlands;; 2 Department of Medical Microbiology and Infectious Diseases, Erasmus University Medical Center, Rotterdam, The Netherlands;; 3 Department of Medical Microbiology, Radboud University Medical Center, Nijmegen, The Netherlands

## Abstract

Controlled human malaria infections (CHMIs) with *Plasmodium falciparum* (*Pf*) parasites are well established. Exposure to five *Pf* (NF54)-infected *Anopheles* mosquitoes results in 100% infection rates in malaria-naïve volunteers. Recently *Pf* clones NF135.C10 and NF166.C8 were generated for application in CHMIs. Here, we tested the clinical infection rates of these clones, using graded numbers of *Pf*-infected mosquitoes. In a double-blind randomized trial, we exposed 24 malaria-naïve volunteers to bites from one, two, or five mosquitoes infected with NF135.C10 or NF166.C8. The primary endpoint was parasitemia by quantitative polymerase chain reaction. For both strains, bites by five infected mosquitoes resulted in parasitemia in 4/4 volunteers; 3/4 volunteers developed parasitemia after exposure to one or two infected mosquitoes infected with either clone. The prepatent period was 7.25 ± 4.0 days (median ± range). There were no serious adverse events and comparable clinical symptoms between all groups. These data confirm the eligibility of NF135.C10 and NF166.C8 for use in CHMI studies.

## INTRODUCTION

Controlled human malaria infections (CHMIs) are a well-accepted tool used since the 1980s for the exploration of immunology and pathophysiology of malaria infections and for evaluation of candidate vaccines and drugs.^1^ The majority of studies have been conducted with 3D7 and its parental strain NF54.^1^ Most volunteers have been infected using five laboratory-reared *Plasmodium falciparum* (*Pf*)–infected *Anopheles* mosquitoes, which reproducibly result in optimal infection rates in malaria-naïve volunteers.^2,3^ Reducing the number of NF54 or 3D7 *Pf*-infected mosquitoes to one or two reduces the infection rate.^4,5^

Given the diversity of *Pf* isolates in the field, the value of CHMI trials can be increased by expanding the portfolio with *Pf* clonal isolates from different geographical origins.^6^ The *Pf* isolate NF54, most likely originates from Africa.^7^ The more recently generated clones are NF135.C10 and NF166.C8, originating from Cambodia and Guinea, respectively. Initial studies with NF135.C10 and NF166.C8 show that these clones are safe and give high infection rates with five infectious mosquitoes (80% [8/10] and 100% [5/5], respectively).^8,9^ However, these clones show more effective hepatocyte invasion and liver merozoite development as compared with NF54, which results in higher first peak of parasitemia and shorter prepatent period.^10^ In order to establish an NF135.C10- or NF166.C8 CHMI model with comparable parasite dynamics as obtained in NF54-infected volunteers, the objective of the current study was to compare infection rates and dynamics of parasitemia as well as clinical manifestations using one, two or five NF135.C10- or NF166.C8- infected mosquitoes.

## MATERIALS AND METHODS

The study is a single-center, double-blind, randomized trial. The flowchart, screening procedures, and inclusion and exclusion criteria were described previously.^10^ Briefly, 24 healthy male and female volunteers, aged 18–35 years, were randomly assigned to six groups and exposed to bites from five mosquitoes of which five, two, or one were infected with either NF135.C10 or NF166.C8. Uninfected mosquitoes were added to preserve blinding of all trial personnel except the staff members preparing and aliquotting infected mosquitoes.

*Anopheles stephensi* mosquitoes were reared and infected according to standardized protocols.^11,12^ The average number of sporozoites per mosquito used in this study was 69,000 for NF135.C10 and 51,000 for NF166.C8. Mosquito feeding was allowed for 10 minutes and infectivity of mosquitoes was assessed after feeding by dissecting salivary glands for the presence of sporozoites. Feeding was repeated until the predefined number of infected bites was reached. Eight volunteers needed two sessions, one needed three sessions (all in the five- or two-mosquito bite groups).

After CHMI, volunteers were followed-up twice daily from day 5 post-infection until two consecutive blood samples were positive by quantitative polymerase chain reaction (qPCR) (≥ 500 parasites/mL), or, when they remained negative, until day 13. At this point, volunteers were treated with atovaquone/proguanil and followed on days 1, 2, 3, and 7 after treatment and on day 35 post-infection. Follow-up was prolonged if volunteers remained qPCR-positive or symptomatic. Throughout the study, high-sensitive troponin T, lactate dehydrogenase (LDH), and platelet counts were measured as safety parameters. The first peak of parasitemia was calculated as the geometric mean of *Pf* parasites per milliliter between day 6.4 and 8.4.^13^

The trial was registered at www.clinicaltrials.gov, identifier NCT02149550. Ethical approval from the Central Committee on Research Involving Human Subjects (NL48704.000.14) was obtained.

All statistical analyses were performed with IBM SPSS statistics for Windows (Version 23.0; IBM Corp., Armonk, NY). Mean prepatent period, first peak of parasitemia, and the frequency of adverse events (AEs) between the groups were assessed by using unpaired *t* test, one‐way analysis of variance (ANOVA), or their nonparametric variants. Adverse events were reported as mild (grade 1), moderate (grade 2), or severe (grade 3).

## RESULTS

In total, 20 of 24 volunteers developed parasitemia; all volunteers exposed to five infected mosquitoes and 3/4 volunteers exposed to one or two infected mosquitoes with either strain (Table 1).

**Table 1:** The number of volunteers developing parasitemia and the prepatent period in days after infection with graded numbers of NF135.C10- or NF166.C8- infected mosquitoes

			No. of infectious bites				*P* value
			Five, two, or one	Five	Two	One	
Strain	NF135.C10	Subjects PCR positive (*n*/total)	10/12	4/4	3/4	3/4	–
		Prepatent period (days (median [range]))	7.5 [7.0–9.0]	7.5 [7.0–9.0]	9.0 [7.5–9.0]	7.0 [7.0–7.0]	0.06
	NF166.C8	Subjects PCR positive (*n*/total)	10/12	4/4	3/4	3/4	–
		Prepatent period (days (median [range]))	7.0 [7.0–11.0]	7.0 [7.0–7.0]	7.0 [7.0–11.0]	9.0 [7.5–9.0]	0.09

Differences between groups were tested using the Kruskal–Wallis method.

**Figure 1.**
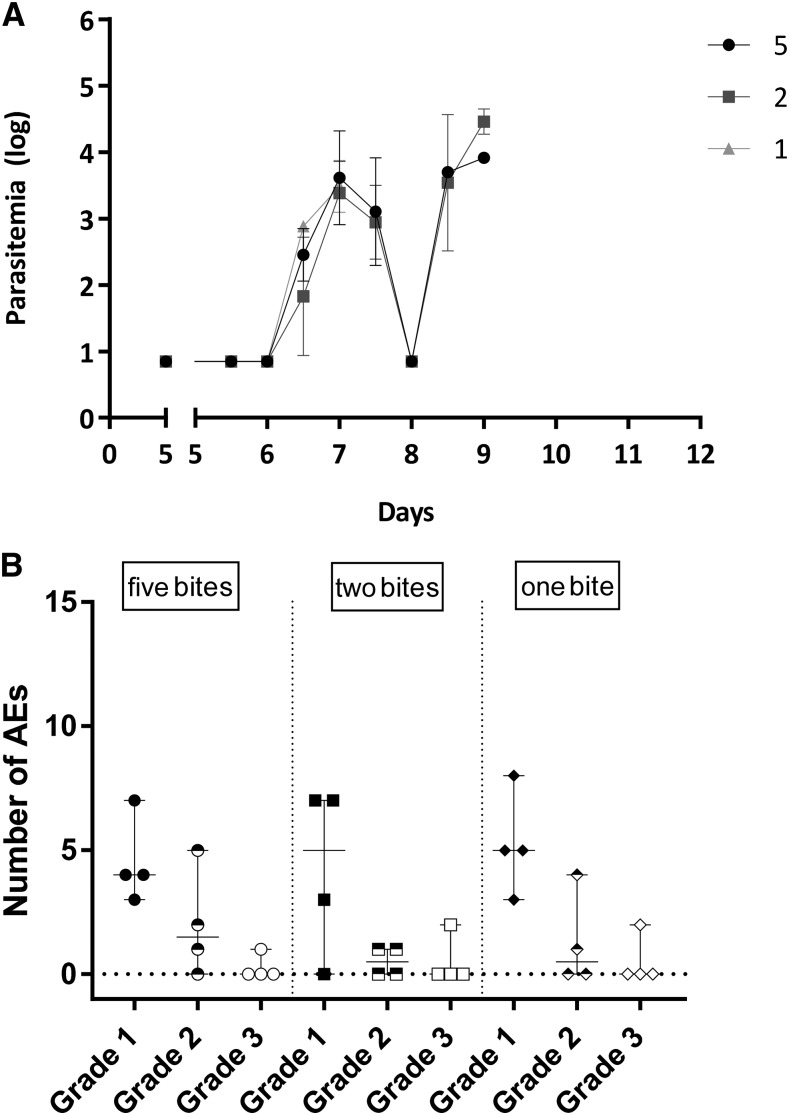
Comparison of one, two, and five NF135.C10-infected mosquito bites (**A**). Kinetics of parasitemia in *Pf*/mL (log) at days post-controlled human malaria infection until qPCR positive (two consecutive samples > 500 *Pf*/mL). Data represent the geometric means and error bars. (**B**). Number of graded adverse events (AE) per volunteer. Symbols represent individual volunteers, and horizontal lines and whiskers represent median and range, respectively. *Pf* = *Plasmodium falciparum*.

The prepatent period in the NF135.C10 groups ranged from 7 to 9 days and did not differ significantly between the dosage groups (median [range] five mosquitoes: 7.5 [7.0–9.0] versus two mosquitoes: 9.0 [7.5–9.0] versus one mosquito: 7.0 [7.0–7.0]; *P* = 0.06, Table 1). There was no difference in first peak of parasitemia in parasites/mL (geometric mean (GM) [95% confidence interval (CI)] five mosquitoes: 2.9 [1.8–4.0], versus two mosquitoes: 2.4 [1.0–3.8], versus one mosquito: 3.0 [2.3–3.7]; *P* = 0.32) (Figure 1A). All parasitemic volunteers experienced one or more AEs. When comparing subjects challenged with either five, two, or one NF135.C10-infected mosquitoes, there were no differences in the total number of grade 1, 2, or 3 AEs (*P* = 0.81, *P* = 0.60, and *P* = 0.87, respectively) (Figure 1B), or the number of solicited AEs (headache, nausea, malaise, chills and myalgia, data not shown). A few unsolicited AEs were reported, two or three per group, all grade 1.

**Figure 2.**
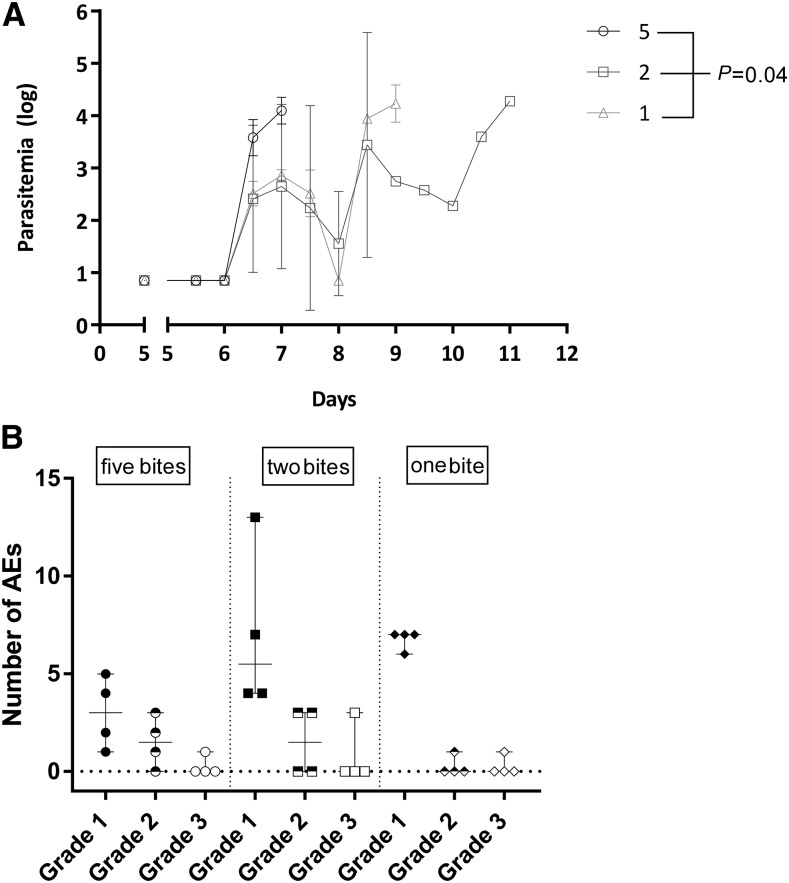
Comparison of one, two, and five NF166.C8-infected mosquito bites (**A**). Kinetics of parasitemia in *Pf*/mL (log) at days post-controlled human malaria infection until qPCR positive (two consecutive samples > 500 *Pf*/mL). Data represent the geometric means and error bars. Significant difference in first peak of parasitemia (geometric mean of day 6.4–8.4) between five, two, or one infected mosquito bites, *P* = 0.04 (**B**). Number of graded adverse events (AEs) per volunteer. Symbols represent individual volunteers, and horizontal lines and whiskers represent median and range, respectively. *Pf* = *Plasmodium falciparum*.

For the NF166.C8 clone, no significant difference was found in the prepatent period between the three dosage groups either (median [range] five mosquitoes: 7.0 [7.0–7.0] versus two mosquitoes: 7 [7.0–11.0] versus one mosquito: 9.0 [7.5–9.0]; *P* = 0.09, Table 1). In contrast to NF135.C10, the first peak of parasitemia was significantly higher in volunteers bitten by higher numbers of NF166.C8-infected mosquitoes (GM [95% CI] five mosquitoes: 3.8 [3.3–4.2], versus two mosquitoes: 2.4 [−0.9 to 5.6], versus one mosquito: 2.2 [1.7–2.7]; *P* = 0.04, Figure 2A). There were no differences in the number of grade 1, 2, or 3 AEs (*P* = 0.11, *P* = 0.33, and *P* = 0.90, respectively) (Figure 2B), nor in the number of solicited AEs between the volunteers challenged with either five, two, or one NF166.C8-infected mosquitoes (data not shown). A few unsolicited AEs were reported, four or six per group, all grade 1.

When comparing all subjects who developed parasitemia with either strain, the prepatent period was similar for NF135.C10 and NF166.C8 (median [range] 7.5 [7.0–9.0] versus 7.0 [7.0–11.0]; *P* = 0.65 see Table 1) as was the height of the first peak of parasitemia (GM [95% CI] NF135.C10: 2.8 [2.4–3.2] parasites/mL versus NF166.C8: 2.9 [2.1–3.6]; *P* = 0.48). In addition, there were no significant differences in the number of grade 1, grade 2, or grade 3 AEs between the two strains (*P* = 0.91, *P* = 0.07, *P* = 0.96).

Upon antimalarial drug treatment, LDH was elevated (> 248 U/L, max 499 U/L) in 10 (50%) malaria-positive volunteers. Platelets were decreased (< 150 × 10^9^/L, min 85 × 10^9^/L) in 11/20. There was no relation with the *Pf*-clone used or the number of bites, and these changes in safety parameters were deemed clinically insignificant. The changes normalized by the end of the study. Throughout the trial hs-troponin T remained lower than 14.0 ng/L in all volunteers.

## DISCUSSION

This study shows that CHMI with five bites of NF135.C10- or NF166.C8-infected mosquitoes results in 100% patent parasitemia, which is comparable with the outcomes of previous CHMIs with NF54, 3D7, or 7G8.^3,14,15^ Lowering the number of infectious bites of both test clones to one or two bites reduced the patency to 75% (3/4 individuals infected), which is similar to previous trials with 3D7-or NF54-infected mosquitoes showing parasitemia in 40–60% of the cases.^4,5^

The kinetics of emerging NF135.C10 parasitemia remained relatively similar irrespective of the number of infected mosquitoes used. Theoretically, a relation with the number of infectious mosquito bites could be expected, as seen in NF166.C8. However, the variance in first peak of parasitemia, in NF135.C10, was large, and these small groups did not allow to find differences. Parasite release from the liver may be subject to variation in sporozoite numbers delivered by the blood-feeding mosquitoes. We previously estimated from CHMI studies that on average 21 sporozoites per mosquito infect hepatocytes, whereas others estimated a release in the range of 1–369 per mosquito.^16,17^ In addition, the probability of infection decreases with a lower sporozoite load (0–1,000) per mosquito.^18^ However, in our studies mosquitoes are infected with > 25,000 sporozoites/mosquito where no such relation exists.^19^

Differences in patency between one, two, or five infected bites suggest there is a strain-independent threshold to be overcome before successful infection occurs which can stochastically result in zero successful events. Such bottlenecks may include the frequency of mosquito probing, the number of sporozoites entering the blood stream, and the number that invade hepatocytes. After hepatocyte invasion, the efficiency of parasite multiplication may differ between strains with a potential higher first peak of parasitemia and correspondingly shorter prepatent periods for NF135.C10 and NF166.C8. This difference in multiplication inside hepatocytes is unlikely related to differences in fitness of these clones, as the IC50 of CSP-antibodies blocking in vitro hepatocyte development is comparable.^9^

Data from this and other studies with NF135.C10- and NF166.C8-infected volunteers showed that there is no increase in the number or severity of AEs compared with NF54-infected volunteers, despite the higher first peak of parasitemia.^10^ The prevalence and severity of AEs will likely further reduce if antimalarial treatment is initiated upon more stringent qPCR cut-off values.^20^

In conclusion, this study with limited numbers of volunteers per group shows that bites of five mosquitoes of NF135.C10 and NF166.C8 of either clone consistently gives 80–100% patent parasitemia in the three studies performed with these heterologous clones. In future CHMI studies using clones NF135.C10 and NF166.C8 and lower numbers of mosquito bites may suffice to achieve blood-stage dynamics which are more similar to NF54, albeit reaching lower infection rates.
